# Development and clinical application of recombinant polyclonal antibodies

**DOI:** 10.3389/fimmu.2026.1849298

**Published:** 2026-08-11

**Authors:** Jiaqi Jin, Guojiang Chen, Fenghao Peng, Jijun Yu, Xinying Li, Ming Yu, Chenghua Liu, Yan Wen, Jiannan Feng, Chunxia Qiao

**Affiliations:** 1 College of Biotechnology, Campus of Jiangsu University of Science and Technology, Zhenjiang, Jiangsu, China; 2 State Key Laboratory of National Security Specially Needed Medicines, Beijing, China

**Keywords:** antibody mixture, mixed master cell bank, recombinant polyclonal antibody, site-specific integration, symphogen

## Abstract

Monoclonal antibodies (mAbs) have transformed the treatment of cancer and immune disorders, but their single-target nature limits efficacy against heterogeneous tumors and mutating pathogens. Recombinant polyclonal antibodies (RPABs)—defined as mixtures of typically 2–25 defined mAbs produced as a single drug substance from one mixed master cell bank—were proposed to combine the epitope breadth of polyclonal antibodies with the manufacturing consistency of mAbs. This review critically analyzes the four RPABs candidates that have entered clinical trials to date (Sym001, Sym004, Sym013, and Sym015), all developed by Symphogen using its proprietary Sympress™ platform. We identify seven interrelated barriers that collectively explain why no RPABs product has yet received regulatory approval: modest efficacy restricted to biomarker-selected subgroups, significant toxicity, pharmacokinetic mismatch among components, instability of mixed cell banks, lack of standardized quality control methods, regulatory uncertainty, and commercial deprioritization after acquisition. Critically, we distinguish between scientific failure (Sym013, discontinued after early termination of its Phase I trial due to tolerability concerns and pharmacokinetic mismatches) and strategic discontinuation (Sym001 and Sym015, which showed clinical signals but were deprioritized for commercial reasons). All clinical and manufacturing data analyzed in this review are derived exclusively from Symphogen’s proprietary Sympress™ platform, as no other RPABs candidate from independent developers has entered clinical trials. We conclude that without independent validation of manufacturing consistency, pharmacokinetic-based component ratio design, and a dedicated regulatory pathway, RPABs face an uncertain future. Recommendations for future development are provided.

## 1 Introduction

The development of antibody drugs has gone through several stages. The concept of therapeutic antibodies originated in 1890, when von Behring and Kitasato demonstrated that sera from immunized animals contained antitoxins capable of neutralizing diphtheria and tetanus toxins, a discovery that earned von Behring the first Nobel Prize in Physiology or Medicine in 1901. Building upon this foundation, Ehrlich proposed the “side-chain theory” in 1897, introducing the “key and lock” model to describe antibody–antigen complementarity. Ehrlich had first used the term “Antikörper” in 1891. In 1975, the hybridoma technology established by Köhler and Milstein enabled the mass production of monoclonal antibodies (mAbs) targeting a single epitope (1). However, early murine monoclonal antibodies have limitations such as high immunogenicity, short half-life, and weak effector function (2). The development of humanization technology (3), phage display and transgenic mouse platforms (4) has promoted the emergence of fully human antibodies.

Nevertheless, the “single-target” nature of monoclonal antibodies still limits their application in complex diseases (5). Polyclonal antibodies have advantages such as multi-epitope coverage, multiple mechanisms of action and high genetic barriers against mutation (6); however, conventional preparation methods suffer from large batch differences, ethical controversies, and complex processes. To balance the controllability of monoclonal antibodies and the breadth of polyclonal antibodies, RPABs have been proposed as a new type of therapeutic agent (7). In this review, we define RPABs as mixtures of 2 to 25 defined monoclonal antibodies, produced as a single drug substance from a single mixed master cell bank. This definition is operational and platform-specific (primarily based on Symphogen’s Sympress™ technology) and should be distinguished from: (i) traditional polyclonal sera (undefined mixtures from immunized animals/humans), (ii) antibody cocktails (components manufactured separately and mixed at final formulation), (iii) bispecific antibodies (single molecule with two binding specificities), and (iv) oligoclonal mixtures (typically 2–4 antibodies from separate cell banks). The term “RPABs” is not yet standardized; some publications use “recombinant polyclonal antibody” interchangeably with “defined antibody mixture.” The component range of 2–25 reflects the current experimental scope of Symphogen’s pipeline; products with fewer or more components could still be considered RPABs as long as they are produced from a single mixed master cell bank as one drug substance. In contrast to RPABs, bispecific antibodies — although also engineered — have established regulatory pathways and multiple approved products (8). A comparison of RPABs, antibody cocktails, and bispecific antibodies is provided in **Table 1**.

**Table 1:** Comparison of recombinant polyclonal antibodies (RPABs), antibody cocktails, and bispecific antibodies.

Feature	RPABs	Antibody cocktail	Bispecific antibody
Production mode	Single mixed cell bank, one batch	Each component produced separately, mixed at final formulation	Single molecule, one batch
Number of components	2–25 full IgGs	Usually 2–4	2–3 binding specificities in one molecule
Batch-to-batch consistency	Potentially high if mixed master cell bank (MCB) ratio is stable; however, risks include clone growth rate drift, expression level changes, and genetic instability	Moderate to high; each component produced separately with own release specs, final mixing ratio can be tightly controlled, but overall consistency depends on multiple manufacturing lines	High
Regulatory path	No dedicated guideline yet	Often regulated as combination product	FDA 2021 guidance exists
Approved products	0	1 (Inmazeb, approved 2020 for Ebola)	>10 (e.g., blinatumomab, amivantamab)

mAb, monoclonal antibody; RPABs, recombinant polyclonal antibodies; FDA, U.S. Food and Drug Administration; MCB, master cell bank.

Symphogen, a Danish company, creatively proposed the concept of recombinant polyclonal antibodies (RPABs). It is currently also the main company that has been dedicated to developing RPABs. In 2001, Symphogen Company took the lead in conducting related research and development, and in 2003, it publicly disclosed its first 25-valent anti-RhD antibody mixture Sym001, marking the official entry of this technology into the industrialization stage. In 2012, Sym001 completed clinical trials to show its safety and effectiveness (9), and then started seeking market opportunities. Over the next decade, the company continued to explore the quality control strategy of RPABs products, and gradually developed a series of RPABs products like Sym001 to at least Sym015. In 2018, a collaboration between Symphogen and Thermo Fisher Scientific was established, and in 2020 Servier (France) announced to have completed the acquisition of Symphogen. This review introduces the key technologies for developing RPABs, the main product pipelines of Symphogen, and the unique quality control methods, which may provide useful information for the development of similar polyclonal drugs.

Unlike previous reviews that focus primarily on Symphogen’s platform, this review critically examines why no RPABs products has yet received regulatory approval.

**Figure 1:**
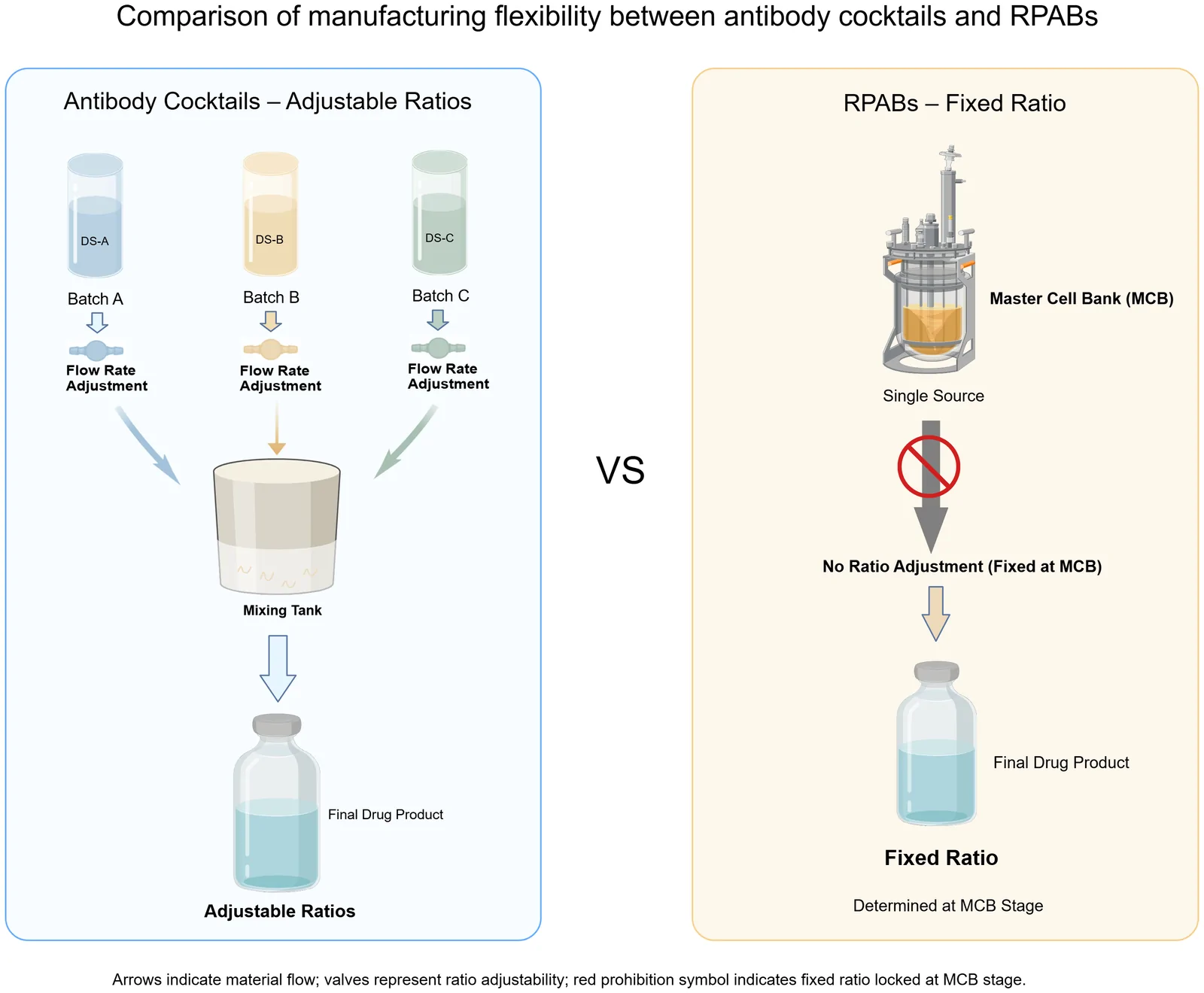
Comparison of manufacturing flexibility between antibody cocktails and RPABs. Left panel (antibody cocktails): three drug substance batches (DS-A, DS-B, DS-C) are independently adjusted via flow rate control valves, then pooled in a mixing tank to achieve adjustable final product ratios. Right panel (RPABs): a single master cell bank (MCB) serves as the sole source, with no ratio adjustment capability, yielding a fixed-ratio final product determined at the MCB stage. Arrows indicate material flow direction. Blue and orange color schemes are used to distinguish the two manufacturing paradigms while ensuring accessibility for color-blind readers. [Figure redrawn using BioGDP.com (11)].

It is also essential to distinguish RPABs from other multi-antibody products that have already reached the market. Inmazeb (atoltivimab/maftivimab/odesivimab-ebgn), approved by the U.S. Food and Drug Administration (FDA) in 2020 for Ebola virus infection, and REGEN-COV (casirivimab/imdevimab), which received Emergency Use Authorization for COVID-19, are both antibody cocktails composed of multiple monoclonal antibodies. However, these approved products are manufactured by producing each component independently in separate cell lines, followed by formulation as a fixed-ratio mixture at the final drug product stage. Each component undergoes its own cell line development, master cell bank establishment, and quality control release, resulting in higher production complexity and cost. In contrast, RPABs are produced as a single drug substance from a single mixed master cell bank, enabling one Good Manufacturing Practice campaign and a single virus clearance validation (10). This manufacturing distinction has three critical regulatory and pharmacokinetic implications. First, antibody cocktails are regulated as combination products and can leverage established regulatory pathways for individual monoclonal antibodies, whereas RPABs lack a dedicated regulatory framework. Second, antibody cocktails offer greater pharmacokinetic flexibility—if one component clears faster than others, its dose can be independently adjusted in the final formulation to maintain the desired *in vivo* ratio. In RPABs, the component ratio is fixed at the master cell bank stage and cannot be adjusted without generating a new MCB, making pharmacokinetic mismatch a more challenging issue. Third, the manufacturing processes differ fundamentally: antibody cocktails require multiple concurrent or sequential production campaigns, while RPABs require only one. These distinctions suggest that the barriers to RPABs approval are not merely generic challenges for multi-antibody therapeutics, but include specific challenges arising from the mixed MCB platform itself. Importantly, the clinical and manufacturing data discussed in the following sections are derived exclusively from Symphogen’s Sympress™ platform, as Symphogen remains the only company that has advanced RPABs candidates into clinical trials. The key differences between the two manufacturing and regulatory pathways are summarized in **Figure 1**.

Literature search strategy: We searched PubMed, Web of Science, ClinicalTrials.gov, and other relevant literature databases for articles published up to the time of manuscript preparation (with updates incorporated through June 2026) using the following keywords and their combinations: “recombinant polyclonal antibodies”, “Symphogen”, “Sym001” (also known as Rozrolimupab), “Sym004”, “Sym013”, “Sym015”, “mixed master cell bank”, “polyclonal master cell bank”, and “antibody mixtures”. Additional relevant publications were identified by manually screening the reference lists of retrieved articles and by reviewing regulatory documents from the FDA and European Medicines Agency websites. We also searched the official websites of Symphogen and Servier for company reports, press releases, and product pipeline updates that are not indexed in conventional academic databases.

## 2 Antibody discovery platforms for RPABs assembly

Prior to Symphogen’s technical platform, the acquisition of antibody variable region sequences—core building blocks of RPABs—did not depend on a single method. Numerous mature strategies were available, each offering unique advantages in generating complementary antibody combinations (12). Antibody discovery strategies are not incompatible with RPABs development principles; theoretically, any monoclonal antibody with defined sequence characteristics can be used to construct RPABs combinations.

### 2.1 Transgenic animal platforms

Fully human monoclonal antibodies represent a core direction in antibody drug development, attributed to their low immunogenicity and high compatibility with human effector functions. Traditional mouse-derived, chimeric, and humanized antibodies carry inherent anti-drug antibody (ADA) induction risks, while *in vitro* display technologies (e.g., phage display) are often limited by insufficient affinity, low expression, and poor druggability. In contrast, genetically modified mouse models with human immunoglobulin gene loci and knocked-out endogenous mouse Ig genes (e.g., XenoMouse, VelocImmune, OmniAb, Trianni) can simulate human antibody production within an intact immune system. Conventional immunization combined with hybridoma technology enables efficient generation of fully human antibodies that have undergone natural affinity maturation and immune tolerance screening. Unlike *in vitro* display, antibodies from this platform undergo *in vivo* negative selection for self-reactivity, hydrophobicity, and aggregation, typically exhibiting superior solubility, thermal stability, and favorable pharmacokinetic profiles (13). Multiple FDA-approved drugs have been developed using this platform, with wide application in oncology and autoimmune diseases (14). While challenges remain (e.g., antibody library diversity, mouse-derived light chain residue leakage), high-throughput sequencing and novel gene editing strategies have continuously optimized antibody quality and coverage, establishing the transgenic mouse platform as a key direction for fully human antibody R&D.

### 2.2 Single B-cell PCR and high-throughput sequencing

This method directly amplifies antibody variable region genes from immune animals or human donors, eliminating the need for hybridoma cell construction and enabling rapid natural antibody library establishment. During the COVID-19 pandemic, Robbiani et al. (15) employed high-throughput B cell single-cell sequencing from recovered individuals to isolate highly effective neutralizing antibodies within weeks. High-throughput immunoglobulin sequencing (Ig-seq) generates hundreds of thousands of antibody sequences and precisely identifies affinity-matured clonal subsets (13). In RPABs development, this method efficiently screens antibodies with high antigenic epitope complementarity, significantly accelerating the screening process and shortening development timelines.

## 3 The technical principles and product pipeline of recombinant polyclonal antibodies

### 3.1 Technical principles and process flow

The core principle of Symphogen’s Sympress™ technology lies in the co-culture of multiple cell lines and the single-batch fabrication of antibody mixtures. To achieve this goal, these cell lines are seeded together at preset ratios to establish a “polyclonal cell bank” (polyclonal master cell bank (pMCB) and polyclonal working cell bank (pWCB)). During fermentation, only one mixed tank needs to be thawed. All cells grow and express together in the same tank, and ultimately, “a single drug substance” (DS) containing all antibodies is harvested at one time. The entire process only requires one GMP procedure and one virus clearance validation to complete the production of antibody mixtures that traditional methods need multiple production lines and multiple rounds of production to achieve (10).

To continuously produce such “scalable” hybrid cell libraries, Symphogen initially relied on its patented Symplex™ antibody discovery platform (16) to identify a series of functional antibodies. This platform employs a single-cell, multiplex overlap-extension and semi-nested PCR approach that amplifies and seamlessly splices natively paired VH-linker-VL fragments from peripheral blood CD19^+^CD38^high^CD45^int^ plasma cells within 15 days without the need for *in vitro* cell culture, generating thousands of Fab clones ready for functional screening (17). To date, numerous antibody discovery technologies, such as transgenic mice and high-throughput sequencing, can also provide antibody genes.

The FRT/Flp-In system employed by Symphogen enables targeted, single-copy integration of antibody genes into predetermined chromosomal hotspots, minimizing position effects and ensuring consistent expression ratios across co-cultured cell lines. In 2006, Symphogen first demonstrated large-scale industrial production using the Sympress™ platform, laying a foundation for the industrialization of recombinant polyclonal antibody production (18). By now, Symphogen has established a product line of recombinant polyclonal antibodies covering multiple fields such as hematological diseases, infectious diseases and solid tumors.

In addition to the FRT/Flp-In system, various site-specific integration strategies have been established in academia for constructing hybrid cell libraries. For instance, Guha & Calos reported that PhiC31 integrase, delivered solely as a protein, can achieve targeted integration at the pseudo-attP site on human chromosome 19q13.31 (19), eliminating the need for plasmid or mRNA mediation (20). The Cre-loxP system allows reversible, high-efficiency editing (21, 22). However, compared to the FRT/Flp-In system, neither has been validated for large-scale multi-lineage mixed MCB production: PhiC31 offers simpler genomic targeting but lacks stability validation in mixed culture, while Cre-loxP requires pre-engineered landing sites and has not been shown to maintain component ratios at scale. Thus, Symphogen’s platform remains the only validated route for industrial RPABs manufacturing.

### 3.2 Product pipeline

#### 3.2.1 Sym001 (Rozrolimupab)

Sym001 is a mixture of 25 fully human IgG1 antibodies targeting RhD antigens. They were derived from B lymphocytes isolated from peripheral blood mononuclear cells (PBMCs) of 8 Rh-negative female donors with high titers of anti-RhD antibodies in their serum, and was used for antibody gene cloning. Antibody profiling analysis revealed a highly conserved VH3–33 gene segment.

Among these 8 women, five had the same VDJ and CDR H3 lengths. Ultimately, 25 antibodies were screened out, covering 99% of RhD variants. Twenty-five independent CHO cell lines were mixed in equal amounts, with a total protein of 300 μg in each bottle. Each line contributed 12 μg of anti-RhD IgG1. Each antibody was expressed, purified and functionally verified to ensure its activity in the mixture. This is the first recombinant polyclonal preparation composed of 25 fully human anti-RhD monoclonal antibodies, used for the treatment of idiopathic thrombocytopenic purpura (ITP) and the prevention of hemolytic disease of the newborn (HDN). Based on preclinical data, *in vitro* experiments have shown that it can coat ≥ 95% of RhD^+^ red blood cells to the phagocytic threshold at a density of 1–2 antibody molecules per red blood cell. After a single administration of 60 μg/kg to cynomolgus macaque, 90% of the circulating RhD^+^ red blood cells were cleared within 24 hours, with a half-life of approximately 21 days, and the effect was comparable to that of Rhophylac^®^ derived from plasma. Phase I/II studies have shown that a single administration of 300 μg/kg can restore the platelet count of 23% of patients to a safe level within 24 hours, with a total response rate of 61.5% on the 7th day, a median duration of 14 days, and only 8% of patients require rescue medication. Without the need for blood transfusion, there was a reversible decrease in hemoglobin, and no death or serious adverse events occurred (9).

Sym001 has completed Phase II development and obtained orphan drug designation from the FDA for immune thrombocytopenia (ITP) treatment. Following Servier’s acquisition of Symphogen in 2020, further clinical development of Sym001 has been contingent on identifying a global collaboration partner, as it was not included in Servier’s core oncology pipeline.

Despite successful Phase II completion and orphan drug status, Sym001 did not advance to Phase III clinical trials. The reasons for this discontinuation are multifactorial and have not been fully disclosed. Potential contributing factors include commercial competition from lower-cost plasma-derived RhIG products, the lack of a clear regulatory pathway for RPABs at the time, and Servier’s post-acquisition strategic focus on oncology. The absence of Phase III development should not be automatically interpreted as evidence of scientific failure, but neither can it be attributed solely to non-scientific factors without direct evidence. From a scientific perspective, it remains unknown whether all 25 antibody components are necessary for full RhD coverage. No published data have examined whether a reduced set of these antibodies could retain equivalent neutralization breadth and potency. Such redundancy analysis could lower manufacturing complexity and improve cost-effectiveness. No updated clinical development has been disclosed since 2012.

#### 3.2.2 Sym004

The construction of Sym004 embodies the concept of “rational combination”. Researchers first used the Symplex technology for antibody screening, obtaining 88 anti-EGFR antibodies from mice. After epitope analysis and functional screening, it was found that mAb992 and mAb1024 bind to the edges of domain III-A and III-C, respectively. They do not compete with each other and form the maximum lattice, thereby increasing the EGFR degradation rate from approximately 30% (with monoclonal antibodies) to >80%. In the A431NS nude mouse model, this 1:1 mixture (50 mg/kg, twice weekly) achieved 100% complete regression and remained without recurrence for 90 days, while cetuximab only showed growth delay. The *in vivo* synergy index reached 2.5, providing dose and mechanism basis for the subsequent Phase I/II clinical trials (23).

All the non-clinical *in vivo* proof-of-concept data of Sym004 are derived from preclinical models. In the A431NS nude mouse xenograft model, a single intraperitoneal injection of 50 mg/kg could completely inhibit the tumor EGFR signal within 3 weeks (the receptor could not be detected by Western blot). Eight out of ten cynomolgus monkeys that were intravenously administered 14 mg/kg weekly developed dose-dependent rashes after the first to third doses, suggesting that the pharmacodynamic biomarker (EGFR inhibition) was rapidly and continuously activated. No data on tumor volume or survival endpoint were disclosed (24). Based on this, Sym004 conducted its first Phase I human trial, which adopted a “3 + 3” dose escalation and expansion design. A total of 62 patients with advanced solid tumors were enrolled, among whom 42 were wild-type RAS metastatic colorectal cancer (mCRC) patients who had been confirmed to be resistant to cetuximab or panitumab. During the dose escalation stage (0.4–12 mg/kg), the maximum tolerated dose was not established. However, based on the cumulative skin and electrolyte toxicity, the recommended extended doses were 9 mg/kg and 12 mg/kg, administered by intravenous infusion weekly. In the expanded cohort, 39 evaluable patients showed an objective response rate of 13% (5 PR cases), a disease control rate of 67%, and a median progression-free survival of 3.3 months. The target lesions shrank in 44% of the patients, and all those who achieved remission were of wild-type KRAS/NRAS/BRAF and had no MET amplification. The toxicity profile was consistent with EGFR inhibition. Grade ≥3 rash and hypomagnesemia were observed in 50% and 21% of patients, respectively, and no treatment-related deaths occurred. Paired biopsy confirmed that the expression of EGFR in tumors and skin was significantly downregulated before the fifth cycle, and Ki-67 decreased simultaneously. This was the first time that the antibody mixture-induced receptor degradation mechanism was verified in humans (25). This study provides a new therapeutic entry point for anti-EGFR-resistant mCRC. In patients with head and neck squamous cell carcinoma (SCCHN), Sym004 also completed a single-arm phase IIa proof-of-concept trial (NCT01417936). Twenty-six patients who had failed previous anti-EGFR treatment received monotherapy with Sym004 at 12 mg/kg once a week. The results showed that the 6-month progression-free survival rate was only 12% (95% CI 1-39%), the median PFS was 82 days, the best efficacy was 50% disease stability, and no objective response was observed. Although skin and tumor biopsies confirmed a significant down-regulation of EGFR expression (p<0.01), and the mechanism was successfully verified, the clinical benefits were limited. Grade ≥3 skin toxicity reached 50%, grade ≥3 hypomagnesemia was 38%, and no new safety signals emerged. This trial became the final clinical data of Sym004 in the field of head and neck cancer, and no further development has been seen since (26). In a multicenter, randomized, phase II clinical trial (Sym004–05 study), a total of 254 patients with KRAS exon 2 wild-type who had previously received anti-EGFR treatment (cetuximab or panitumab) and developed acquired resistance were included in the study and randomly divided into three groups: Sym004–12 mg/kg per week (Group A), 6 mg/kg per week after 9 mg/kg loading (Group B), or the investigator-selected treatment (Group C). The primary endpoint was overall survival (OS). The median OS in the ITT population was 7.9, 10.3, and 9.6 months, respectively, which did not reach a statistical difference. However, in the “triple-negative” subgroup screened by ctDNA molecules (with no mutations in RAS/BRAF/EGFR ECD), the median OS in group Sym004 B was significantly prolonged to 12.8 months, which was 5.5 months longer than that in group C (7.3 months), suggesting that Sym004 may have potential benefits in the molecular selection population (27).

However, a Phase III trial in unselected patients with refractory metastatic colorectal cancer (mCRC) was withdrawn prior to enrollment, and Sym004 was subsequently deprioritized from Servier’s active pipeline following its acquisition of Symphogen in 2020. Further development of Sym004 will likely require a collaborative partner.

Sym004 exhibited the designed EGFR degradation activity; however, this activity did not translate to improved survival outcomes in unselected patients. Even with biomarker-guided patient selection, the clinical benefit remained modest, and no objective responses were observed in squamous cell carcinoma of the head and neck (SCCHN). These findings indicate that receptor degradation alone is insufficient to achieve meaningful clinical efficacy, highlighting the need for integrated biomarker strategies early in RPABs development.

#### 3.2.3 Sym013

Sym013 is a mixture composed of six humanized IgG1 monoclonal antibodies, targeting non-overlapping epitopes on the HER family (HER1/EGFR, HER2, HER3) (28). The screening of Pan-HER began by immunizing mice with soluble recombinant EGFR, HER2, HER3 antigens or cancer cells overexpressing these receptors. Subsequently, based on its core Symplex antibody discovery platform, approximately 50 candidate antibodies were initially obtained for each receptor, totaling approximately 150 antibodies. Then, these antibodies were paired and combined in multiple cancer cell lines to evaluate their ability to synergistically inhibit tumor growth. Eventually, six antibodies were screened out, among which the EGFR antibodies (1277 and 1565) targeted two non-overlapping epitopes of receptor Domain III. HER2 antibodies (4384 and 4517) bind to Domain III and Domain IV respectively, while HER3 antibodies (5038 and 5082) both bind to Domain I but with different epitopes. Functional verification shows that these antibodies can synergistically inhibit the proliferation of cancer cells *in vitro* and significantly down-regulate the levels of target proteins, which is superior to using any one antibody alone. Thus, a Pan-HER mixture is formed (29).

A total of 32 patients with advanced epithelial tumors were enrolled in the first-in-human phase I trial of Sym013, with a median age of 58 years. 31% of them had colorectal cancer, and 34% had received EGFR/HER2 targeted therapy. The dose escalation started from 1 mg/kg and was attempted up to 15 mg/kg in Q2W. Due to the occurrence of grade ≥2 oral mucositis, rash and diarrhea at 4 mg/kg, the dosing interval was forced to be changed from Q1W to Q2W, and systemic hormones, antihistamines and other compulsory pretreatment were added at the 9 mg/kg level. In terms of safety, 100% of the patients experienced treatment-related adverse events, 81% had skin toxicity, 75% had gastrointestinal toxicity, 44% had infusion reactions, 22% had grade ≥3 toxicity, and 13% of the patients permanently discontinued the drug due to toxicity. Ultimately, it was concluded that 15 mg/kg Q2W exceeded the maximum tolerated dose, and the MTD was not officially established. In terms of efficacy, among the 29 evaluable patients, only 1 rectal cancer patient achieved confirmed partial response (PR, lasting 39.1 weeks), 12 patients had stable disease (SD) for ≥6 weeks, and 4 patients had SD for ≥16 weeks. The median treatment cycle was only 2 cycles. Pharmacokinetics showed that the exposure to the six antibodies increased with the increase of dose. However, EGFR antibodies were cleared quickly, and their trough concentrations were generally lower than those of HER2/HER3 antibodies. Low-titer anti-drug antibodies were detected in 16% of the patients. The trial was prematurely terminated for commercial reasons and has not entered the expansion period (30).

The six-antibody pan-HER mixture induced high toxicity (22% grade ≥3), leading to early trial termination. Differential clearance profiles between EGFR and HER2/HER3 antibodies demonstrated that *in vitro*-optimized antibody ratios may not be sustained *in vivo*. Future RPABs development should prioritize matching the half-lives of individual antibody components to maintain optimal *in vivo* efficacy and safety. Whether Fc engineering could correct the clearance mismatch remains unexplored.

#### 3.2.4 Sym015

Two humanized IgG1 antibodies targeting non-overlapping epitopes of MET, named Hu9006 and Hu9338 respectively, are used for the treatment of advanced solid tumors with abnormally activated MET and have now entered phase Ia/IIa clinical trials (31).

BALB/c, C57BL/6 and C3H mice were immunized with different combinations of MET digested by HCT116 cells and trypsin, recombinant MET and recombinant MET combined with HGF. Isolate the B-cell suspension from the spleen of immunized mice. Single-cell clonal antibodies were cloned using Symplex technology. After screening out high-affinity antibodies, humanization treatment was carried out and they were expressed as complete IgG1 antibodies. Finally, a 1:1 mixture composed of two antibodies (Hu9006 + Hu9338), namely Sym015, was selected (32).

The first-in-human phase Ia/IIa trial conducted in patients with advanced solid tumors demonstrated good safety and preliminary anti-tumor activity. As of April 2019, a total of 51 patients (with an average age of 61 years) were enrolled, among whom 12 received treatment in the dose escalation stage and 39 entered the dose expansion stage. The study adopted a dosing regimen of once every two weeks (q2w). Four doses of 6, 12, 18 and 24 mg/kg were evaluated in the dose escalation phase. The final recommended Phase II dose (RP2D) was a loading dose of 18 mg/kg followed by a maintenance dose of 12 mg/kg. Most of the patients had non-small cell lung cancer (NSCLC, n=14) and gastric cancer (n=12). Among the NSCLC patients, 6 cases had MET amplification (METAmp), 7 cases had MET exon 14 deletion (METEx14D), and 1 case had both variations simultaneously. Among the 14 NSCLC patients, one patient with MET amplification (6.8 copies detected by NGS) achieved partial response (PR, with a 53% reduction in tumor size), and the response lasted for 80 weeks. Among the remaining patients, the majority achieved disease stability (SD), and the tumor shrinkage in some patients was between 20% and 30% (33). The ESMO 2020 update focused the efficacy analysis on the population with MET/CEP7≥5 or MET exon14 deletion (n=42), increasing the ORR to 25%, the DCR to 65%, and the median PFS to 4.1 months, indicating that high MET amplification is a key biomarker for predicting benefits. The pharmacokinetic curve is dose-dependent. The half-life after the first administration is 7 to 10 days, and there is no accumulation toxicity after multiple administrations. Dynamic monitoring of ctDNA further confirmed that an early decrease in MET copy number was significantly associated with longer PFS. Overall, Sym015 is well tolerated and achieves durable remission in tumors with high MET amplification.

In addition, public company disclosures indicate multiple ongoing RPABs research projects. As reported by Fierce Biotech (April 2020), Sym009 is being developed by Roche’s Genentech division for the treatment of Staphylococcus aureus bacteremia. No detailed clinical data have been disclosed since then, and the project’s current status remains unclear (34).

Potential of RPABs in infectious diseases. Beyond oncology, RPABs may offer distinct advantages in the treatment of rapidly mutating pathogens, where the ability to target multiple conserved epitopes may offer a higher genetic barrier to immune escape than monoclonal antibodies. This mechanism is conceptually analogous to convalescent plasma or polyclonal sera, but with the critical distinction that RPABs provide a defined, reproducible composition. For viruses such as SARS-CoV-2, influenza, or HIV, the emergence of escape variants frequently limits the long-term efficacy of single monoclonal antibodies, a challenge that antibody cocktails like REGEN-COV were designed to address. However, cocktails produced from separate cell lines require independent manufacturing and regulatory approval for each component, whereas RPABs might theoretically achieve similar or broader neutralization breadth from a single mixed MCB. Sym009, which was being developed by Roche/Genentech for *Staphylococcus aureus* bacteremia, represents an early effort in this direction, though no clinical data have been disclosed. Future development in infectious diseases would likely require careful selection of antibody components targeting non-overlapping, conserved epitopes, combined with preclinical models that account for viral evolution. Whether the RPABs format offers a practical advantage over antibody cocktails in this context requires further investigation in relevant preclinical models and, ultimately, clinical studies.

Sym015 achieved durable clinical responses exclusively in patients with high MET amplification (MET/CEP7≥5), with no meaningful responses observed in those with low MET amplification or isolated MET exon14 deletion. These results confirm that RPABs exhibit strong target dependency; the absence of Phase III data further indicates that Sym015’s continued development will require a commercial partnership. A comprehensive overview of reported RPABs candidates is provided in **Table 2**. All clinical-stage RPABs originate from Symphogen, reflecting the technical barrier of mixed-cell-bank establishment.

**Table 2:** Reported RPABs candidates and their clinical development status.

Product name	Target(s)	No. of components	Indication(s)	Development stage (highest)	Key clinical findings	Safety issues	PK observation	Current status
Sym001 (Rozrolimupab)	RhD antigens	25	Idiopathic thrombocytopenic purpura (ITP) and prevention of hemolytic disease of the newborn (HDN)	Completed Phase II	Coats RhD^+^ RBCs; 61.5% response; rapid clearance	Reversible Hb decrease; no deaths or SAEs	t_1_/_2_ ≈ 21 days (cynomolgus monkey)	Phase II complete; no Phase III; needs partner
Sym004	EGFR	2	Anti-EGFR resistant mCRC, SCCHN	Phase II (Phase III withdrawn)	13% ORR in mCRC; no OS benefit; biomarker subgroup potential	Rash, hypomagnesemia; no treatment-related deaths	Not reported	Deprioritized after Servier acquisition; needs partner
Sym013	Pan-HER (EGFR/HER2/HER3)	6	Advanced epithelial tumors	Early terminated Phase I	1 PR; differential clearance; short treatment	22% grade≥3; 13% discontinuation of medication; skin/GI toxicity	EGFR Abs cleared faster than HER2/HER3 Abs	Terminated early for commercial reasons
Sym015	MET	2	Advanced MET-activated solid tumors	Phase Ia/IIa	25% ORR in high MET amp; durable PR.	Well tolerated; no accumulation toxicity.	t_1_/_2_ 7–10 days; dose-dependent	Awaits partnership for further progress
Sym009	Staphylococcus aureus	/	Staphylococcus aureus bacteremia	Not disclosed	/	/	/	Project progress unknown

RPABs, recombinant polyclonal antibodies; ORR, objective response rate; DCR, disease control rate; PFS, progression-free survival; PR, partial response; SD, stable disease; SAE, serious adverse event; mCRC, metastatic colorectal cancer; SCCHN, squamous cell carcinoma of the head and neck; ND, not disclosed. Clinical trial identifiers: Sym004-05; Sym013 Phase I; Sym015 Phase Ia/IIa.

To date, Symphogen is the only company that has advanced RPABs into clinical trials. No other RPABs candidates from independent developers have been reported in the clinical stage, reflecting the technical and regulatory barriers discussed below.

### 3.3 Why has no RPABs product been approved?

Despite twenty years of research and multiple trials, there is currently no RPABs product approved for market launch. Based on the above clinical data, we identified seven interrelated barriers that collectively explain this translation gap.

These seven barriers can be grouped into four hierarchical levels according to the nature of the challenge they represent. Conceptual barriers (a–c) concern the therapeutic limitations intrinsic to the RPABs design paradigm, including variable efficacy in unselected populations, tolerability concerns, and the requirement for predictive biomarkers—challenges that arise from the multi-component nature of RPABs themselves. Platform barriers (d–e) arise from technical challenges associated with the mixed MCB manufacturing process, specifically pharmacokinetic mismatch among components and CMC complexity; these platform-level issues often exacerbate the conceptual limitations described above. Regulatory barriers (f) reflect the lack of dedicated guidelines, which forces developers to adopt customized quality control schemes without a clear precedent. Commercial barriers (g) reflect strategic and market factors, including portfolio rebalancing and competition from alternative products, which have affected the development continuity of several RPABs candidates. This hierarchical framework clarifies whether each barrier is intrinsic to the RPABs concept, specific to the Sympress™ platform, or external to the technology itself. The following analysis applies this framework to each barrier in turn.

Before analyzing the individual barriers, it is critical to distinguish between scientific failures and strategic discontinuations among the four RPABs candidates. Sym013 was discontinued because of tolerability concerns (grade ≥3 adverse events in 22% of patients) and pharmacokinetic mismatches among its six components—highlighting a key limitation of the RPABs design approach. In contrast, Sym001 and Sym015 showed promising clinical signals (a 61.5% response rate for Sym001 in ITP; a 25% ORR in a subset of patients with MET-amplified NSCLC for Sym015) but were deprioritized after Servier’s acquisition of Symphogen in 2020. For Sym001, competition from lower-cost plasma-derived RhIG products also contributed to its commercial challenges. Sym004 occupies an intermediate position: it demonstrated biomarker-dependent activity but did not show a statistically significant improvement in overall survival in unselected populations, and its Phase III trial was withdrawn. This distinction is important because the barriers to RPABs approval are heterogeneous; addressing them requires differentiated strategies depending on whether the underlying challenge is scientific, technical, or commercial.

#### 3.3.1 Clinical efficacy [conceptual level]

Objective responses have been observed only in biomarker-selected subsets. Sym004 achieved only 13% ORR in unselected mCRC and no objective response in SCCHN. Sym015 showed 25% ORR exclusively in the MET/CEP7≥5 subgroup. Sym013 produced only one partial response in 29 evaluable patients.

#### 3.3.2 Toxicity [conceptual level]

Severe toxicity can affect clinical development. Sym013 caused grade 3 or higher adverse events in 22% of patients, and overall adverse events in 100% of patients, leading to premature trial termination. Sym004 induces grade ≥ 3 rash in up to 50% of patients and grade ≥ 3 hypomagnesemia in 21-38% of patients.

#### 3.3.3 Biomarker selection [conceptual level]

The modest efficacy of Sym004 and Sym015 in unselected populations underscores the need for predictive biomarkers. For Sym004, only patients with RAS/BRAF/EGFR ECD wild-type tumors showed potential benefit. For Sym015, high MET amplification (MET/CEP7 ≥5) was required for responses. Future RPABs development must integrate biomarker strategies from early stages.

#### 3.3.4 Pharmacokinetic mismatch [platform level]

Sym013 demonstrated that *in vitro*-optimized antibody ratios may not be sustained *in vivo*: EGFR antibodies cleared faster than HER2/HER3 antibodies, disrupting the intended mixture composition and contributing to the trial’s early termination.

#### 3.3.5 CMC complexity [platform level]

As discussed in Section 3.4, mixed MCBs face potential instability of component ratios and post-translational heterogeneity (35). The absence of industry-wide standardized analytical methods and the lack of independent verification of Symphogen’s QC workflows further complicate regulatory acceptance.

#### 3.3.6 Regulatory uncertainty [regulatory level]

No dedicated guideline exists for RPABs. The FDA 2021 bispecific antibody guidance explicitly excludes polyclonal products, and the European Pharmacopoeia has no monograph for RPABs formulations. This regulatory gap forces developers to adopt customized QC schemes, complicating cross-project comparisons and regulatory review.

#### 3.3.7 Commercial and strategic factors [commercial level]

Following Servier’s acquisition of Symphogen in 2020, Sym001, Sym004, and Sym015 were deprioritized from the active pipeline. Sym001 faced competition from cheaper plasma-derived RhIG products. The lack of a global collaboration partner has stalled further development (36).

#### 3.3.8 Design optimization potential

**Figure 2:**
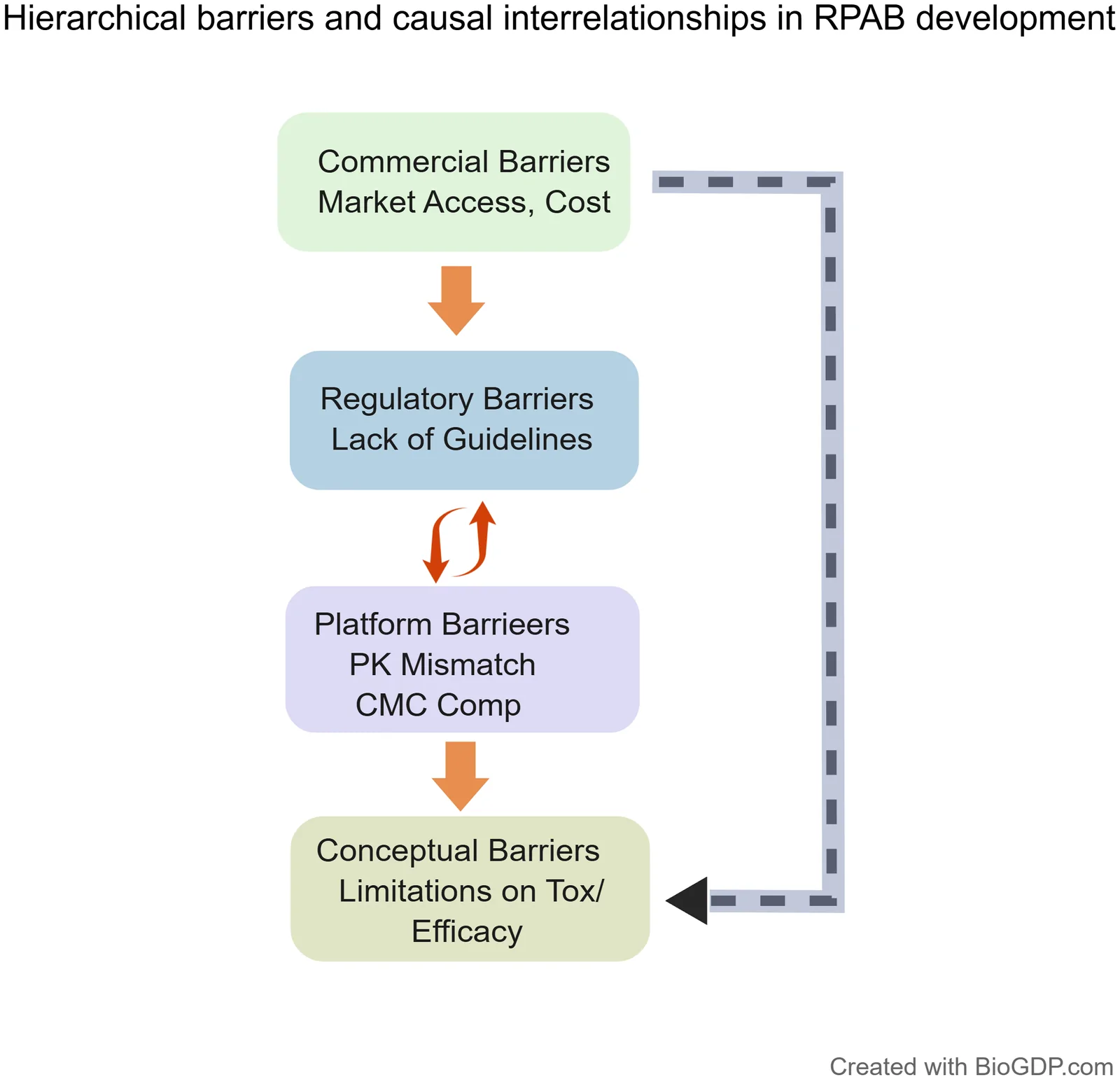
Hierarchical barriers and causal interrelationships in RPAB development. The four-tier framework comprises, from bottom to top, Conceptual, Platform, Regulatory, and Commercial barriers. Arrows denote causal interactions: a bidirectional arrow between Regulatory and Platform barriers indicates mutual reinforcement between the lack of regulatory guidelines and CMC complexity; a unidirectional arrow from Platform to Conceptual barriers illustrates how PK mismatch drives limitations in toxicity and efficacy evaluation; a dashed curved arrow represents the market feedback loop that feeds back into conceptual design. A pastel color palette is applied to ensure accessibility for readers with color vision deficiencies. [Figure redrawn using BioGDP.com (11)].

Beyond the barriers listed above, several scientific questions remain unexplored. For Sym001, it is unknown whether all 25 components are necessary or if a smaller subset could achieve equivalent RhD coverage; component redundancy analysis could reduce manufacturing complexity. For Sym013, whether the differential clearance of EGFR versus HER2/HER3 antibodies can be corrected by Fc engineering (e.g., half-life extension mutations) has not been investigated. Addressing such questions could improve future RPABs designs and should be part of the translational agenda. The hierarchical relationships among these barriers are summarized in **Figure 2**.

## 4 Component analysis and quality control strategies of recombinant polyclonal antibodies

The biological function of RPABs is largely dependent on component diversity and the utilization of non-overlapping epitopes. Consequently, quality control must prioritize precise analysis, verification, and batch-to-batch consistency of these structural attributes. The quality control strategies developed by Symphogen highlight key differences between RPABs and mAbs, though independent validation of these approaches remains limited.

### 4.1 Epitope design and Component analysis

Rozrolimupab (Sym001) is composed of 25 unique fully human IgG1 monoclonal antibodies. These antibodies jointly target multiple different epitopes on the RhD red blood cell antigen, forming “epitope saturation coverage”, which greatly reduces the risk of immune escape caused by variations in a single epitope; Sym004 is composed of two antibodies targeting different domains of the epidermal growth factor receptor (EGFR) (recognized by Futuximab and Modotuximab respectively) in a 1:1 ratio (37). Both antibodies recognize different epitopes of EGFR, thereby avoiding competitive binding (25). Structural biology research has confirmed that the epitopes bound to these two antibodies do not overlap spatially, thus they can simultaneously bind to EGFR to form efficient receptor cross-linking.

Before delving into epitope design, it is worth noting that chromatographic separation techniques have long served as the analytical backbone for monoclonal antibody (mAb) characterization, including size-exclusion chromatography (SEC), ion-exchange chromatography (IEX), and reversed-phase chromatography (RPLC) for assessing purity, charge variants, and post-translational modifications (38–40). For recombinant polyclonal antibodies (RPABs), which consist of multiple distinct mAb entities, such orthogonal analytical methods become even more critical to ensure accurate component identification and epitope mapping.

Epitope analysis is critical for ensuring that each antibody targets a different antigenic epitopes. Symphogen’s platform uses Epitope Binning technology to divide candidate antibodies according to the epitope regions they bind to, ensuring these epitopes are distinct and non-overlapping. For example, when screening Sym004, it was confirmed through this method that mAb992 and mAb1024 bound to non-overlapping epitopes within EGFR domain III, respectively, and were not competitive (23). Similarly, the six antibodies in Pan-HER were also clearly demarcated and targeted different domains on EGFR, HER2 and HER3 (29). This rational epitope selection lays a structural foundation for subsequent functional collaboration.

This multivalent, non-overlapping epitope design provides the structural basis for RPABs synergy. Therefore, verifying these attributes is a logical starting point for quality control, as discussed below.

### 4.2 Consistency of component control

To ensure that each batch of products can accurately replicate the above design structure, Symphogen Company has established a quality control strategy centered on structural analysis. Component consistency is the cornerstone of RPABs quality control, ensuring that the types and relative proportions of all antibody components in each batch of products comply with the preset standards. Take the Sym001 drug as an example. The “identity file” and release strategy of the 25 component antibodies in Sym001 go through the following stages.

Cell Banking Strategy for RPABs. Standard monoclonal antibody production relies on a hierarchical cell bank structure consisting of three tiers: primary cell bank (PCB), master cell bank (MCB), and working cell bank (WCB) (International Council for Harmonisation, 1997). The key innovation of RPABs lies in the MCB stage: unlike conventional MCBs derived from a single cloned cell line, RPABs MCB is a specific mixture formed by combining multiple recombinant cell lines in pre-designed proportions (41). Once the hybrid MCB passes qualification testing, each batch of WCB prepared from this integrated MCB maintains consistent antibody component characteristics. This allows RPABs products to be classified as a single drug substance rather than a multi-component mixture, which fundamentally distinguishes RPABs from antibody mixtures— the latter requires independent production and quality control for each component (18).

There are many potential risks and practical difficulties in the application of hybrid master cell banks. Although this mode can theoretically maintain a constant antibody ratio, there are differences in the proliferation rate and protein expression ability of different CHO cell clones (42), and long-term cultivation is prone to antibody proportion deviation. At the same time, monoclonal strains may undergo gene recombination and gene silencing, further altering the composition of the cellular mixed system (35).

The glycosylation, charge variants, aggregates, and other post-translational modification characteristics of each cloned expression product are different, and the traits will continue to fluctuate during the culture stage. Co cultivation with multiple clones will release host proteins and residual DNA, greatly increasing the difficulty of impurity removal. Compared to monoclonal antibodies, the difficulty of evaluating product quality consistency is significantly higher after adjusting the large-scale production process.

Symphogen addressed some of these risks by demonstrating ratio stability over >80 generations and using orthogonal analytical methods (LC-MS, peptide mapping) to monitor consistency. However, independent validation on other RPABs is lacking. Therefore, ratio stability, genetic stability, and product quality attributes must be verified empirically for each new RPABs products.

First, the antibody mixture is subjected to reduction and alkylation to separate heavy and light chains, thereby eliminating the structural complexity of intact IgG. The 25 light chains, each with a unique theoretical mass differing by at least 13 Da, naturally form a “mass barcode”. Under optimized reversed-phase chromatography combined with high-resolution mass spectrometry, all 25 light chains can be precisely resolved, allowing for unambiguous identification of each component— a critical step in ensuring the quality and consistency of RPABs.

For quantification, a characteristic peptide-based relative quantitation method was developed using extracted ion chromatography normalized to an internal standard. The method was fully validated according to ICH Q2(R1), demonstrating good linearity (R² ≥ 0.98), acceptable recovery (69–134%, mostly 90–110%), and precision (RSD 1.9–10.2% for intermediate precision). Industrial validation across four 200 L GMP batches showed high consistency, with a fingerprint deviation of less than 0.4 % from conventional cation-exchange chromatography. This marked the first release of a recombinant polyclonal antibody product using mass spectrometry (43). Subsequently, Kristensen et al. (44) developed an automated two-step SMART enzymatic digestion workflow that minimizes missed cleavages and hydrophobic peptide carry-over, a common issue in traditional peptide mapping. By replacing C18 with a C4 reversed-phase column, they improved reproducibility, sequence coverage, and data quality, demonstrating the potential of the multi-attribute method (MAM) for GMP release and stability testing of RPABs (44). Multi-laboratory validation of MAM for complex biologics has recently been reported (45).

In contrast to traditional monoclonal antibody (mAb) quality control strategies, which predominantly focus on a single molecular entity and rely on well-established analytical techniques including size-exclusion chromatography (SEC), cation-exchange chromatography (CEX), and capillary electrophoresis-sodium dodecyl sulfate (CE-SDS) (46), quality control of recombinant polyclonal antibodies (RPABs) demands the simultaneous monitoring of the relative proportions of multiple component entities while upholding an equivalent level of analytical stringency. The analytical approaches outlined above, namely liquid chromatography-mass spectrometry (LC-MS) and peptide mapping, are directly adapted from the well-validated analytical toolbox for mAb characterization (43, 47). Cumulative evidence has demonstrated that the RPABs format can be effectively quality-controlled using orthogonal analytical techniques that are comparable in rigor and reliability to those routinely employed for monoclonal antibody quality control.

Additionally, Symphogen entered into a collaborative partnership with Thermo Fisher Scientific starting from 2018, with the primary goal of developing mass spectrometry-based analytical workflows tailored for the characterization of complex antibody mixtures, thereby providing robust technical support for the aforementioned QC strategies. This collaboration contributed to the development of the multi-attribute method (MAM) for RPABs characterization.

### 4.3 Functional characterization and validation

Functional validation is a systematic assessment of the biological activity and therapeutic potential of recombinant polyclonal antibodies through *in vivo*, *in vitro* and synergistic therapy studies. Given that recombinant polyclonal antibodies are composed of multiple monoclonal antibodies, if pairwise matching verification is conducted one by one, both the experimental throughput and cost will increase exponentially, which is not feasible in practical operation. Therefore, the current strategy adopts a “holistic hybrid, *in vitro* - *in vivo* integrated functional assessment” model: polyclonal antibodies are treated as a single entity. Their overall efficacy is first screened through an *in vitro* activity matrix, and then their overall *in vivo* function is verified using animal models. By integrating functional consistency assurance, this QC strategy streamlines scalability and enhances screening efficiency.

For efficacy verification, the research of Sym004 has established a complete three-level evidence chain of “cell - animal - molecule”. Mutations in the extracellular domain (ECD) of the epidermal growth factor receptor (EGFR) are well-documented to mediate resistance to targeted therapeutic agents. Notably, Sym004, a recombinant polyclonal antibody (RPABs) formulation, has been shown to overcome such EGFR ECD mutation-induced drug resistance. *In vitro* functional assays demonstrated that Sym004 specifically binds to and blocks all clinically relevant EGFR ECD mutants—including S492R, G465R, R451C, and K467T—at the EGFR-Y1068 phosphorylation site, which is accompanied by the down-regulation of the AKT/ERK signaling pathway and a significant reduction in the clonogenic potential of mutant cells. In nude mouse xenograft models established with EGFR mutant cell lines (DCR7-S492R and LIM1215-G465R), intraperitoneal administration of Sym004 at a dose of 40 mg/kg resulted in continuous regression of S492R-harboring tumors and a delay in G465R-driven tumor growth by more than 2 weeks. Furthermore, this therapeutic intervention led to almost complete degradation of intratumoral EGFR, a reduction in the proliferation marker Ki-67 to 2%, and a significant upregulation of the apoptotic marker cleaved caspase-3 (48). To verify whether Sym004 can overcome cetuximab resistance, researchers used cetuximab-resistant (CtxR) NCI-H226 cells and the “induction-then change” mouse model. Sym004 at 20 mg/kg twice a week could achieve 67% objective growth delay in large tumors >1000 mm³. Along with the almost complete degradation of tumor EGFR, the positive rate of Ki-67 decreased from 18% to 2%, while cleaved caspase-3 increased accordingly. The activity of MAPK/RSK1 was widely downregulated (49).

In the radiation sensitization study, a similar three-level approach was followed. First, four NSCLC/HNSCC cell lines were selected *in vitro* and pretreated with 10 μg/mL Sym004 for 24 hours. Then, the colony formation experiment was used. The repair proteins such as DNA-PK and BRCA1 were detected by parallel γH2AX focal count, Annexin V/PI apoptotic flow cytometry and Western blot to confirm the accumulation of DNA damage and the inhibition of repair. H226 or SCC1483 cells were subcutaneously inoculated into BALB/c nude mice *in vivo*. When the tumor diameter reached 6–8 mm, they were randomly divided into 4 groups (IgG control, single drug, single release, combination), and Sym004 (ip, 1.6–80 mg/kg) was administered according to the preset protocol. Twice a week and/or radiotherapy (18 Gy or 6 × 3 Gy each time), measure the tumor volume with a caliper every day, draw the growth curve at the endpoint and calculate the regrowth delay time; Tumor tissues were taken for IHC/fluorescence verification of EGFR down-regulation, γH2AX increase and DNA-PK nuclear translocation reduction to achieve a complete chain of evidence from molecular mechanism to animal efficacy (50). The two studies jointly demonstrated that the degrading EGFR antibody mixture can both break through monoclonal antibody resistance and work in synergy with radiotherapy to amplify DNA damage.

Pan-HER (EGFR/HER2/HER3 three-target six-antibody) achieved a maximum inhibition rate of >75% against EGFR, HER2 or HER3-dependent strains in the screening of 104 cell lines covering 13 cancer types, which was significantly better than that of single-target antibodies (P<0.001). Among the four PDX models (ovarian ST024, colorectal CRC095, lung ST599, pancreatic ST191), tumor regression occurred in three quarters of the Pan-HER group. Among them, the tumor volume of the ST191 model decreased by 90% on the 20th day (p<0.01). Cetuximab or trastuzumab only showed growth retardation (29). In order to surpass the trastuzumab and pertuzumab dual-drug regimen, Symphogen used its Symplex technology to systematically screen more than 1,300 bis-tri-4 antibody combinations (51). The study first used the HER2 extracellular domain four-segment chimeric antigen (human-mouse substitution) to classify 156 independent antibodies into four epitope loci of subdomains I, II, III, and IV. Subsequently, eight cell lines such as HER2-amplified gastric cancer NCI-N87 and breast cancer SK-BR-3 were used as panels to determine the proliferation inhibition of WST-1 over 4 days, and the synergy was quantified using a nonlinear mixed model. The results showed that the 1:1:1 tripartite mixture (referred to as “tripartite mixture” in the text) that simultaneously combined subdomains I (mAb 4387), II (mAb 4382), and IV (mAb 4517) had the broadest activity. The HER2 high expression/EGFR high co-expression model is superior to trastuzumab + pertuzumab. This mixture is rapidly degraded by receptor clustering-endocytosis-lysosomes, blocking HER2-EGFR/HER3 cross-phosphorylation and inducing complement-dependent cytotoxicity (CDC); In transplanted tumors of OE19 and NCI-N87 mice, the 50 mg/kg biweekly regimen achieved 100% complete remission with no recurrence within 8 weeks (51). Mechanically, both Pan-HER and HER2 trisclonal antibodies achieve an internalization rate of over 80% within 4 hours and a degradation rate of ≥60% within 24 hours through “multi-point cross-linking”, and block EGFR/HER3 cross-phosphorylation. Pan-HER simultaneously inhibits downstream pERK and pAKT for more than 48 hours, avoiding signal rebound within 24 hours after single-target blocking.

### 4.4 Limitations in independent validation and standardization

Although RPABs exhibit remarkable advantages in their design philosophy, they harbor critical inherent flaws that pose substantial obstacles to their clinical translation, commercialization, and regulatory approval. A fundamental challenge lies in the absence of a dedicated regulatory framework tailored to RPABs. The U.S. Food and Drug Administration (FDA) 2021 guidance on bispecific antibodies clarifies that bispecific antibodies are defined as single molecular entities and thus not subject to fixed-dose combination regulations; critically, this guidance explicitly excludes polyclonal antibody products from its scope. Similarly, while the European Pharmacopoeia provides general monographs for monoclonal antibody products, no dedicated monograph has been established to date for recombinant polyclonal antibody formulations, resulting in a prominent regulatory gap. This gap necessitates the adoption of customized quality control (QC) schemes, which must be independently developed by the developers of each RPABs candidate, thereby complicating cross-project comparisons and substantially increasing the complexity of regulatory review processes. Kumar et al. noted that although the regulatory framework for polyclonal antibody (pAb)-based therapeutic products is gradually evolving, there remains a lack of standardized analytical methodologies to ensure batch-to-batch consistency—a critical prerequisite for regulatory approval. The team further emphasized that the intrinsic molecular complexity and heterogeneity of RPABs pose significant challenges to comprehensive product characterization, rigorous quality control, and efficient regulatory review (52).

The absence of a dedicated RPABs guideline does not mean an absent regulatory path. Existing ICH guidelines (Q5A–Q5E, Q6B) remain applicable by analogy. For example, ICH Q5A (viral safety) and Q5D (cell banks) apply directly to mixed MCBs. ICH Q6B (specifications for biotechnological products) provides a framework for setting release and stability criteria, although it was designed for single molecular entities. The key regulatory question is whether RPABs should be treated as a single drug substance (like a mAb) or as a combination product (like a fixed-dose cocktail). The FDA 2021 bispecific antibody guidance explicitly excludes polyclonal products, leaving this question unresolved.

Regulators are likely to ask specific questions during RPABs review, including: (i) component control – how identity and relative abundance of each antibody are measured and controlled; (ii) potency – whether bioassays measure the contribution of each component or only the mixture as a whole; (iii) comparability – how manufacturing changes affect each component and how consistency is demonstrated; (iv) product consistency – acceptance criteria for batch-to-batch variation in component ratios; (v) immunogenicity – risk assessment for each component, including anti-drug antibodies that may cross-react; and (vi) clinical contribution – evidence that each antibody in the mixture adds therapeutic value. These questions have not yet been systematically addressed for any RPABs candidate.

Compounding this regulatory gap is the absence of industry-wide standardized analytical methods for RPABs. The United States Pharmacopeia (USP) Chapter <129> provides well-validated procedures for the purity assessment of monoclonal antibodies, including SEC-HPLC, capillary electrophoresis, and CE-SDS, yet no analogous guidance exists for polyclonal products derived from mixed master cell banks (MCBs). The lack of orthogonal, independently validated reference methods represents a major bottleneck to regulatory acceptance of RPABs. Consistent with this challenge, Kumar et al. argued that pAbs have been underutilized in therapeutic applications due to the absence of robust, standardized analytical tools,” a statement that directly reflects the current predicament faced by RPABs developers (52).

Another critical limitation is the lack of independent verification of Symphogen’s published detection methodologies. While the company has released several reports describing LC-MS-based detection methods (43, 44) and mixed MCB manufacturing processes (41), no third-party laboratory has independently reproduced these results using a different RPABs products. This lack of external validation—a well-established standard for platform-wide acceptance—raises unresolved questions regarding the generalizability and robustness of these QC strategies outside Symphogen’s proprietary development environment. The broader biopharmaceutical industry has recognized that “complex multi-component mixtures” require advanced analytical strategies to ensure product quality and safety; however, for RPABs specifically, progress toward the establishment of standardized, publicly available reference methods has been sluggish. First, the LC-MS quantification method developed for Sym001 shows a recovery range of 69–134%, with most values falling within 90–110% but with notable outliers (43). While acceptable for exploratory characterization, this level of variability is wider than what is typically expected for routine GMP release of monoclonal antibody products, suggesting that further optimization may be needed before the method can be routinely applied to lot-release. Second, Symphogen demonstrated ratio stability over >80 generations (41), which provides valuable preliminary evidence. However, this does not constitute long-term stability data across the entire product lifecycle. The potential impact of cumulative genetic drift, clonal outgrowth, or gene silencing over extended manufacturing campaigns remains to be fully characterized, particularly given that CHO cell lines are known to exhibit instability over prolonged culture (35). Third, and most notably, all published QC data for RPABs—including LC-MS methods, peptide mapping workflows, and mixed MCB stability studies—have been generated exclusively by Symphogen. To date, no third-party laboratory has independently reproduced these results using a different RPABs product. This lack of external validation, which is a standard expectation for platform-wide analytical methods, represents a notable gap that currently hinders broader regulatory acceptance of the proposed QC workflows. Recent multi-laboratory validation efforts for the multi-attribute method (MAM) in the context of monoclonal antibodies provide a useful precedent (45), and analogous efforts specifically for RPABs would be valuable.

To address the aforementioned gaps, it is imperative for stakeholders across the biopharmaceutical industry, scientific research community, and regulatory authorities to collaborate closely in order to jointly establish platform-level reference methods, unified quality standards, and clear regulatory pathways that are specifically applicable to this novel class of biological products (46, 52).

### 4.5 Key quality attributes for RPABs

A structured quality control strategy for RPABs should address component identity and relative abundance (e.g., by LC-MS), binding specificity and epitope coverage (e.g., by epitope binning), glycosylation and Fc-related attributes, charge and size variants, aggregates and fragments, host cell proteins and residual DNA, potency assays, batch release and stability testing, reference standards, and comparability after manufacturing changes. Published work on Sym001 and Sym004 has exemplified some of these aspects, but industry-wide standardization remains lacking. The corresponding analytical methods for these quality attributes are listed in **Table 3**.

**Table 3:** Analytical methods for RPABs quality control.

Quality attribute	Analytical method(s) as described in the manuscript
Component identity & abundance	LC-MS (light chain mass barcoding, characteristic peptide quantitation)
Epitope coverage	Epitope binning
Charge variants	Cation-exchange chromatography (CEX)
Aggregates/fragments	SEC, CE-SDS
Host cell proteins & residual DNA	ELISA, qPCR
Potency	Cell-based bioassay (ADCC, CDC, proliferation inhibition)
Comparability	Multi-attribute method (MAM)

LC-MS, liquid chromatography-mass spectrometry; SEC, size-exclusion chromatography; CE-SDS, capillary electrophoresis-sodium dodecyl sulfate; CEX, cation-exchange chromatography; ELISA, enzyme-linked immunosorbent assay; qPCR, quantitative polymerase chain reaction; ADCC, antibody-dependent cell-mediated cytotoxicity; CDC, complement-dependent cytotoxicity; MAM, multi-attribute method.

**Table 4** provides a qualitative comparison of RPABs and monoclonal antibodies in selected disease contexts.

**Table 4:** Comparison of antibody formats in selected disease contexts.

Disease context	RPABs	Monoclonal antibodies	Antibody cocktails	Multi-specific antibodies
High-heterogeneity solid tumor	May offer advantages (multi-epitope)	Standard of care for some targets	Moderate benefit	Limited valency
Fast-mutating pathogen	Potential benefit (broad neutralization)	Often limited by immune escape	Broader than mAbs	Can be designed

This table provides a qualitative comparison of theoretical advantages in selected disease contexts and is not based on head-to-head clinical trial data.

## 5 Summary and outlook

Recombinant polyclonal antibody (RPABs) technology represents an innovative therapeutic paradigm that successfully integrates the epitope breadth of polyclonal antibodies with the batch-to-batch controllability of monoclonal antibodies (mAbs). Compared with traditional antibody cocktails, RPABs can incorporate a greater number of component antibodies and achieve broader epitope coverage, thereby potentially reducing the risk of immune escape—a critical concern in treating pathogens or tumors with high mutational potential. Symphogen has demonstrated the feasibility of this therapeutic model through its proprietary core technology platform and systematic quality control (QC) strategies, laying a foundation for the translational development of RPABs. However, as discussed earlier, dedicated regulatory frameworks for RPABs remain undefined, analytical methodologies lack industry-wide standardization, and independent validation of developer-specific QC workflows is still insufficient. These interconnected challenges collectively account for the fact that no RPABs product has yet obtained marketing approval worldwide.

Distinct from antibody cocktails, which require separate production and independent QC for each component—leading to increased operational complexity and cost-RPABs are manufactured as a single drug substance from a mixed master cell bank (MCB), simplifying production processes while maintaining component homogeneity. Compared with bispecific or trispecific antibodies, which typically target a limited number of epitopes to achieve synergistic effects, RPABs offer superior epitope coverage, an advantage that may be most relevant in the treatment of highly mutable pathogens or heterogeneous tumors, although this has not been clinically validated. The absence of approved RPABs products to date reflects a confluence of technical (QC standardization deficits), regulatory (lack of dedicated frameworks), and/or commercial factors (e.g. Servier’s strategic refocus on oncology), none of which invalidate the inherent value of the RPABs concept; instead, these challenges underscore the urgent need for platform-level standardization and coordinated regulatory pathways to facilitate translational progress.

To establish RPABs as a mature therapeutic platform, future work must provide: (i) clinical validation in larger, randomized trials; (ii) industry-wide standardized analytical methods; (iii) dedicated regulatory guidance; and (iv) independent verification of manufacturing consistency.

## References

[1] Kaplon H Chenoweth A Crescioli S Reichert JM . Antibodies to watch in 2022. MAbs. (2022) 14:2014296. doi: 10.1080/19420862.2021.2014296 35030985 PMC8765076

[2] Buss NA Henderson SJ McFarlane M Shenton JM de Haan L . Monoclonal antibody therapeutics: history and future. Curr Opin Pharmacol. (2012) 12:615–22. doi: 10.1016/j.coph.2012.08.001 22920732

[3] Almagro JC Fransson J . Humanization of antibodies. Front Biosci. (2008) 13:1619–33. doi: 10.2741/2786 17981654

[4] Murphy AJ Macdonald LE Stevens S Karow M Dore AT Pobursky K . Mice with megabase humanization of their immunoglobulin genes generate antibodies as efficiently as normal mice. Proc Natl Acad Sci U S A. (2014) 111:5153–8. doi: 10.1073/pnas.1324022111 24706856 PMC3986188

[5] Wang C Hesketh EL Shamorkina TM Li W Franken PJ Drabek D . Antigenic structure of the human coronavirus OC43 spike reveals exposed and occluded neutralizing epitopes. Nat Commun. (2022) 13:2921. doi: 10.1038/s41467-022-30658-0 35614127 PMC9132891

[6] Lu H Liu Y Song Y Chen L Zhang L Li R . Neutralizing antibodies vs. Viruses: interacting mechanisms and escape tactics. Microorganisms. (2025) 13:2199. doi: 10.3390/microorganisms13092199 41011531 PMC12472874

[7] Haurum JS . Recombinant polyclonal antibodies: the next generation of antibody therapeutics? Drug Discov Today. (2006) 11:655–60. doi: 10.1016/j.drudis.2006.05.009 16793535

[8] Labrijn AF Janmaat ML Reichert JM Parren PWHI . Bispecific antibodies: a mechanistic review of the pipeline. Nat Rev Drug Discov. (2019) 18:585–608. doi: 10.1038/s41573-019-0028-1 31175342

[9] Robak T Windyga J Trelinski J von Depka Prondzinski M Giagounidis A Doyen C . Rozrolimupab, a mixture of 25 recombinant human monoclonal RhD antibodies, in the treatment of primary immune thrombocytopenia. Blood. (2012) 120:3670–6. doi: 10.1182/blood-2012-06-438804 22915649

[10] Rasmussen SK Næsted H Müller C Tolstrup AB Frandsen TP . Recombinant antibody mixtures: Production strategies and cost considerations. Arch Biochem Biophys. (2012) 526:139–45. doi: 10.1016/j.abb.2012.07.001 22820097

[11] Jiang S Li H Zhang L Mu W Zhang Y Chen T . Generic Diagramming Platform (GDP): a comprehensive database of high-quality biomedical graphics. Nucleic Acids Res. (2025) 53:D1670–6. doi: 10.1093/nar/gkae973 39470721 PMC11701665

[12] Beerli RR Rader C . Mining human antibody repertoires. MAbs. (2010) 2:365–78. doi: 10.4161/mabs.12187 20505349 PMC3180084

[13] Mompo SM Gonzalez-Fernandez A . Antigen-specific human monoclonal antibodies from transgenic mice. Methods Mol Biol. (2019) 1904:253–91. doi: 10.1007/978-1-4939-8958-4_11 30539474

[14] Giusti RM Shastri KA Cohen MH Keegan P Pazdur R . FDA drug approval summary: panitumumab (Vectibix). Oncologist. (2007) 12:577–83. doi: 10.1634/theoncologist.12-5-577 17522246

[15] Robbiani DF Gaebler C Muecksch F Lorenzi JCC Wang Z Cho A . Convergent antibody responses to SARS-CoV-2 in convalescent individuals. Nature. (2020) 584:437–42. doi: 10.1038/s41586-020-2456-9 32555388 PMC7442695

[16] Meijer PJ Andersen PS Haahr Hansen M Steinaa L Jensen A Lantto J . Isolation of human antibody repertoires with preservation of the natural heavy and light chain pairing. J Mol Biol. (2006) 358:764–72. doi: 10.1016/j.jmb.2006.02.040 16563430

[17] Meijer PJ Nielsen LS Lantto J Jensen A . Human antibody repertoires. Methods Mol Biol. (2009) 525:261–77. doi: 10.1007/978-1-59745-554-1_13 19252857

[18] Wiberg FC Rasmussen SK Frandsen TP Rasmussen LK Tengbjerg K Coljee VW . Production of target‐specific recombinant human polyclonal antibodies in mammalian cells. Biotechnol Bioeng. (2006) 94:396–405. doi: 10.1002/bit.20865 16596663

[19] Ahmadi M Mahboudi F Akbari Eidgahi MR Nasr R Nematpour F Ahmadi S . Evaluating the efficiency of phiC31 integrase-mediated monoclonal antibody expression in CHO cells. Biotechnol Progr. (2016) 32:1570–6. doi: 10.1002/btpr.2362 27604579

[20] Guha TK Calos MP . Nucleofection of phiC31 integrase protein mediates sequence-specific genomic integration in human cells. J Mol Biol. (2020) 432:3950–5. doi: 10.1016/j.jmb.2020.04.019 32339531

[21] Foster MP Benedek MJ Billings TD Montgomery JS . Dynamics in Cre-loxP site-specific recombination. Curr Opin Struct Biol. (2024) 88:102878. doi: 10.1016/j.sbi.2024.102878 39029281 PMC11616326

[22] Kawabe Y Makitsubo H Kameyama Y Huang S Ito A Kamihira M . Repeated integration of antibody genes into a pre-selected chromosomal locus of CHO cells using an accumulative site-specific gene integration system. Cytotechnology. (2012) 64:267–79. doi: 10.1007/s10616-011-9397-y 21948097 PMC3386388

[23] Koefoed K Steinaa L Soderberg JN Kjaer I Jacobsen HJ Meijer PJ . Rational identification of an optimal antibody mixture for targeting the epidermal growth factor receptor. MAbs. (2011) 3:584–95. doi: 10.4161/mabs.3.6.17955 22123060 PMC3242845

[24] Skartved NJ Jacobsen HJ Pedersen MW Jensen PF Sen JW Jorgensen TK . Preclinical pharmacokinetics and safety of Sym004: a synergistic antibody mixture directed against epidermal growth factor receptor. Clin Cancer Res. (2011) 17:5962–72. doi: 10.1158/1078-0432.CCR-11-1209 21825041

[25] Dienstmann R Patnaik A Garcia-Carbonero R Cervantes A Benavent M Roselló S . Safety and activity of the first-in-class Sym004 anti-EGFR antibody mixture in patients with refractory colorectal cancer. Cancer Discov. (2015) 5:598–609. doi: 10.1158/2159-8290.Cd-14-1432 25962717

[26] Machiels JP Specenier P Krauss J Dietz A Kaminsky MC Lalami Y . A proof of concept trial of the anti-EGFR antibody mixture Sym004 in patients with squamous cell carcinoma of the head and neck. Cancer Chemother Pharmacol. (2015) 76:13–20. doi: 10.1007/s00280-015-2761-4 25952795

[27] Montagut C Argilés G Ciardiello F Poulsen TT Dienstmann R Kragh M . Efficacy of sym004 in patients with metastatic colorectal cancer with acquired resistance to anti-EGFR therapy and molecularly selected by circulating tumor DNA analyses. JAMA Oncol. (2018) 4;e175245. doi: 10.1001/jamaoncol.2017.5245 29423521 PMC5885274

[28] Reddy TP Choi DS Anselme AC Qian W Chen W Lantto J . Simultaneous targeting of HER family pro-survival signaling with Pan-HER antibody mixture is highly effective in TNBC: a preclinical trial with PDXs. Breast Cancer Res. (2020) 22:48. doi: 10.1186/s13058-020-01280-z 32414394 PMC7227035

[29] Jacobsen HJ Poulsen TT Dahlman A Kjær I Koefoed K Sen JW . Pan-HER, an antibody mixture simultaneously targeting EGFR, HER2, and HER3, effectively overcomes tumor heterogeneity and plasticity. Clin Cancer Res. (2015) 21:4110–22. doi: 10.1158/1078-0432.Ccr-14-3312 25908781

[30] Berlin J Tolcher AW Ding C Whisenant JG Horak ID Wood DL . First-in-human trial exploring safety, antitumor activity, and pharmacokinetics of Sym013, a recombinant pan-HER antibody mixture, in advanced epithelial Malignancies. Invest New Drugs. (2022) 40:586–95. doi: 10.1007/s10637-022-01217-7 35113285

[31] Poulsen TT Grandal MM Skartved NJO Hald R Alifrangis L Koefoed K . Sym015: A highly efficacious antibody mixture against MET-amplified tumors. Clin Cancer Res. (2017) 23:5923–35. doi: 10.1158/1078-0432.CCR-17-0782 28679766

[32] Grandal MM Havrylov S Poulsen TT Koefoed K Dahlman A Galler GR . Simultaneous targeting of two distinct epitopes on MET effectively inhibits MET- and HGF-driven tumor growth by multiple mechanisms. Mol Cancer Ther. (2017) 16:2780–91. doi: 10.1158/1535-7163.MCT-17-0374 28802255

[33] Camidge DR Janku F Bueno AM Catenacci DV Lee J Lee SH . A phase Ia/IIa trial of Sym015, a MET antibody mixture, in patients with advanced solid tumours. Ann Oncol. (2019) 30:v610–1. doi: 10.1093/annonc/mdz260.012

[34] Butler MS Paterson DL . Antibiotics in the clinical pipeline in October 2019. J Antibiot (Tokyo). (2020) 73:329–64. doi: 10.1038/s41429-020-0291-8 32152527 PMC7223789

[35] Dahodwala H Lee KH . The fickle CHO: a review of the causes, implications, and potential alleviation of the CHO cell line instability problem. Curr Opin Biotechnol. (2019) 60:128–37. doi: 10.1016/j.copbio.2019.01.011 30826670

[36] Schuhmacher A Gassmann O Hinder M . Changing R&D models in research-based pharmaceutical companies. J Transl Med. (2016) 14:105. doi: 10.1186/s12967-016-0838-4 27118048 PMC4847363

[37] Sanchez-Martin FJ Bellosillo B Gelabert-Baldrich M Dalmases A Canadas I Vidal J . The first-in-class anti-EGFR antibody mixture sym004 overcomes cetuximab resistance mediated by EGFR extracellular domain mutations in colorectal cancer. Clin Cancer Res. (2016) 22:3260–7. doi: 10.1158/1078-0432.CCR-15-2400 26888827

[38] Deol S Matsuda Y Hiruta Y . Current advances in separation chemistry for antibody purification and analysis. Anal Sci. (2025) 41:653–66. doi: 10.1007/s44211-025-00748-2 40126861

[39] Jafarzadeh Chehraghi E Akbarzadehlaleh P Shamsasenjan K . Purification of monoclonal antibodies using chromatographic methods: increasing purity and recovery. Adv Pharm Bull. (2025) 15:27–45. doi: 10.34172/apb.43967 40636302 PMC12235372

[40] Song X Tian H Jing R Liu K Xu K Guo L . Navigating the Frontier: Advances in monoclonal antibody purity control. Protein Expression Purif. (2025) 232:106725. doi: 10.1016/j.pep.2025.106725 40345637

[41] Frandsen TP Naested H Rasmussen SK Hauptig P Wiberg FC Rasmussen LK . Consistent manufacturing and quality control of a highly complex recombinant polyclonal antibody product for human therapeutic use. Biotechnol Bioeng. (2011) 108:2171–81. doi: 10.1002/bit.23166 21495017

[42] Pilbrough W Munro TP Gray P . Intraclonal protein expression heterogeneity in recombinant CHO cells. PloS One. (2009) 4:e8432. doi: 10.1371/journal.pone.0008432 20037651 PMC2793030

[43] Persson P Engstrom A Rasmussen LK Holmberg E Frandsen TP . Development of mass spectrometry based techniques for the identification and determination of compositional variability in recombinant polyclonal antibody products. Anal Chem. (2010) 82:7274–82. doi: 10.1021/ac101175w 20690610

[44] Kristensen DB Ørgaard M Sloth TM Christoffersen NS Leth-Espensen KZ Jensen PF . Optimized multi-attribute method workflow addressing missed cleavages and chromatographic tailing/carry-over of hydrophobic peptides. Anal Chem. (2022) 94:17195–204. doi: 10.1021/acs.analchem.2c03820 36346901 PMC9753059

[45] Shipman J Oyugi M Marzan TA Geerlof-Vidavsky I Kirkpatrick D Zhu H . A multi-laboratory, multi-platform analysis of the multi-attribute method. Pharmaceuticals. (2025) 18:1613. doi: 10.3390/ph18111613 41304860 PMC12655225

[46] Yang Y Li M Xu G Ni Y Guo L Yu C . Recent advances and applications of capillary electrophoresis-sodium dodecyl sulfate for characterization and fragment identification of monoclonal antibodies and their derivatives. Analyst. (2026) 151:93–104. doi: 10.1039/d5an00918a 41416511

[47] Hess S Carvalho SB Gomes RA Pfenninger A Fischer M Strotbek M . Multi attribute method implementation using a High Resolution Mass Spectrometry platform: From sample preparation to batch analysis. PloS One. (2022) 17:e0262711. doi: 10.1371/journal.pone.0262711 35085302 PMC8794205

[48] Sanchez-Martin FJ Dalmases A Bellosillo B Argiles G Gelabert M Cañadas I . Abstract 2149: The anti-EGFR antibody mixture Sym004 overcomes acquired resistance to cetuximab in colorectal cancer. Cancer Res. (2016) 76:2149. doi: 10.1158/1538-7445.Am2016-2149 26888827

[49] Iida M Brand TM Starr MM Li C Huppert EJ Luthar N . Sym004, a novel EGFR antibody mixture, can overcome acquired resistance to cetuximab. Neoplasia. (2013) 15:1196–206. doi: 10.1593/neo.131584 24204198 PMC3819635

[50] Huang S Peet CR Saker J Li C Armstrong EA Kragh M . Sym004, a novel anti-EGFR antibody mixture, augments radiation response in human lung and head and neck cancers. Mol Cancer Ther. (2013) 12:2772–81. doi: 10.1158/1535-7163.MCT-13-0587 24130052 PMC3960925

[51] Pedersen MW Jacobsen HJ Koefoed K Dahlman A Kjaer I Poulsen TT . Targeting three distinct HER2 domains with a recombinant antibody mixture overcomes trastuzumab resistance. Mol Cancer Ther. (2015) 14:669–80. doi: 10.1158/1535-7163.MCT-14-0697 25612619

[52] Kumar S Tini A Tengattini S Rinaldi F Calleri E Massolini G . Polyclonal antibody therapeutics: analytical innovations and regulatory perspectives for addressing heterogeneity challenges. Anal Chem. (2026) 98:5828–42. doi: 10.1021/acs.analchem.5c06531 41712472 PMC12961642

